# Chromobox 7/8 serve as independent indicators for glioblastoma *via* promoting proliferation and invasion of glioma cells

**DOI:** 10.3389/fneur.2022.912039

**Published:** 2022-08-11

**Authors:** Zong-Qing Zheng, Gui-Qiang Yuan, Na-Ling Kang, Qian-Qian Nie, Guo-Guo Zhang, Zhong Wang

**Affiliations:** ^1^Department of Neurosurgery and Brain and Nerve Research Laboratory, The First Affiliated Hospital of Soochow University, Suzhou, China; ^2^Department of Neurosurgery, Changshu Second People's Hospital, Suzhou, China; ^3^Liver Center, The First Affiliated Hospital, Fujian Medical University, Fujian, China

**Keywords:** glioblastoma (GBM), chromobox (CBX), prognosis, multi-omics, proliferation, invasion

## Abstract

**Background:**

The chromobox family, a critical component of epigenetic regulators, participates in the tumorigenesis and progression of many malignancies. However, the roles of the CBX family members (CBXs) in glioblastoma (GBM) remain unclear.

**Methods:**

The mRNA expression of CBXs was analyzed in tissues and cell lines by Oncomine and Cancer Cell Line Encyclopedia (CCLE). The differential expression of CBXs at the mRNA level was explored in The Cancer Genome Atlas (TCGA) and Chinese Glioma Genome Atlas (CGGA) databases with the “beeswarm” R package. The protein expression of CBXs in GBM was further examined on Human Protein Atlas (HPA). The correlations between CBXs and IDH mutation and between CBXs and GBM subtypes were investigated in the TCGA portal and CGGA database with the “survminer” R package. The alteration of CBXs and their prognostic value were further determined *via* the cBioPortal and CGGA database with the “survival” R package. The univariate and multivariate analyses were performed to screen out the independent prognostic roles of CBXs in the CGGA database. Cytoscape was used to visualize the functions and related pathways of CBXs in GBM. U251 and U87 glioma cells with gene intervention were used to validate the role of CBX7/8 in tumor proliferation and invasion. Proliferation/invasion-related markers were conducted by Western blot and immunostaining.

**Results:**

CBXs presented significantly differential expressions in pan-cancers. CBX2/3/5/8 were upregulated, whereas CBX6/7 were downregulated at mRNA level in GBM of TCGA and CGGA databases. Similarly, high expression of CBX2/3/5 and low expression of CBX6/8 were further confirmed at the protein level in the HPA. CBX2/6/7 were positively correlated with IDH mutation and CBX1/2/4/5/8 were closely related to GBM subtypes. CBX7 and CBX8 presented the independent prognostic factors for GBM patient survival. GO and KEGG analyses indicated that CBXs were closely related to the histone H3-K36, PcG protein complex, ATPase, and Wnt pathway. The overexpression of CBX7 and underexpression of CBX8 significantly inhibited the proliferation and invasion of glioma cells *in vivo* and *in vitro*.

**Conclusion:**

Our results suggested that CBX7 and CBX8 served as independent prognostic indicators that promoted the proliferation and invasion of glioma cells, providing a promising strategy for diagnosing and treating GBM.

## Introduction

Glioblastoma (GBM) is the most aggressive and lethal brain tumor in adults (1). Despite new advances in surgical resection combined with chemoradiotherapy (2), the prognosis of GBM patients remains dismal with only about 14.6 months, and <10% of patients could survive over 5 years (3). The poor prognosis is closely related to the tumor progression, invasion, and recurrence of GBM. Thus, more efforts were urgent to reveal these potential molecular mechanisms in GBM, which would provide more effective targets for GBM treatment.

Recent studies have demonstrated that epigenetic dysregulation played a critical role in oncogenesis (4), suggesting that targeting the epigenetic mechanism may be hopeful for tumor treatment in the future (5). As a classical family of epigenetic regulatory complexes, the chromobox family members (CBXs) function as regulatory proteins to promote and enhance elements of genes (6). This family includes eight members with two subtypes that suppressed histone 3 lysine trimethylation at residues K9 and K27 (H3K9me3 and H3K27me3), respectively. CBX1, CBX3, and CBX5 belong to the heterochromatin protein 1 (HP1) complex group, which interprets H3K9me3 marks by H3K9 methyltransferases. Besides, CBX2, CBX4, CBX6, CBX7, and CBX8 are Polycomb Repressive Complex 1 (PRC1) group that recognizes H3K27me3 marks. The CBX family could regulate histone proteins *via* interacting with post-translational modifications in the N-terminal amino acid sequences, which contributed to the tumorigenesis in breast (7), colorectal (8), kidney (9), hepatocellular (10), and lung cancer (11, 12). In malignant brain tumors, previous studies showed that CBX3 could promote the growth of glioma cells and displayed a prognostic role for glioma patients *in vitro* and *in vitro* (13). The CCNE1 (14) and YAP/TAZ (15) pathways regulated by CBX7 could promote the proliferation or migration of glioma cells *in vitro*. CBX8 also presented a differential expression in glioma tissues (16), which exhibited a prognostic value for predicting glioma patients' survival (17). All the above studies focused on the role of a single member of CBX family in glioma. However, the comprehensive function of other CBXs family members in glioma, especially in GBM, was not fully explored.

This study conducted comprehensive multi-omics analyses of the CBX family in The Cancer Genome Atlas (TCGA) and Chinese Glioma Genome Atlas (CGGA) databases, including genomics, transcriptomics, and proteomics. Moreover, each prognostic value was also analyzed *via* Kaplan-Meier, univariate, and multivariate survival analyses using R software with the “survival” and “survminer” packages. Clinical GBM classification, including IDH mutation and GBM subtypes, was also explored in GBM samples with differential expressions of CBXs. Finally, we conducted further experiments *in vivo* and *in vitro* to examine the effect of the CBX family on the proliferation and invasion of GBM, which may provide novel prognostic and therapeutic targets for the treatment of this devastating disease.

## Materials and methods

### Ethics statement

Human GBM samples were collected from the patients with fully informed consent in accordance with the World Medical Association's Declaration of Helsinki. The study was approved by the Ethics Committee of the First Affiliated Hospital of Soochow University. Animal experiments were conducted following the International Association of Veterinary Editors guidelines. The experimental protocols were also approved by the Ethics Committee of the First Affiliated Hospital of Soochow University.

### Oncomine

The Oncomine database (www.oncomine.org) is used to explore the differential expressions of CBXs at mRNA level in various cancers (18). We focused on the expression of CBXs in Brain and CNS cancers. The specific fold changes of the CBX family in glioma were also counted (fold changes should be beyond 1.5 and gene ranks should be in the top 10%).

### Cancer cell line encyclopedia database

Cancer cell lines provided independent information on CBXs in various cancers *in vitro*. We further explored and compared the expressions of CBXs between brain cancer cells and other cancer cells in CCLE (https://portals.broadinstitute.org/ccle/), an mRNA database including 1,457 human cancer cell lines (19).

### TCGA and CGGA GBM databases

TCGA and CGGA databases were both used in our research. The mRNA expression of the CBX family in GBM of TCGA was obtained from UALCAN, a TCGA database with 31 cancers RNA-seq and clinical data (http://ualcan.path.uab.edu) (20). The TCGA portal, a website based on the TCGA database (http://tumorsurvival.org/) (21), was used to analyze the relationship between the CBXs expression and GBM subtypes. Furthermore, we downloaded 218 WHO IV primary GBM samples with their clinical information from CGGA, a Chinese database with over 2,000 samples and 1,018 mRNA sequencings with clinical data (www.cgcg.org.cn) (22–25), as shown in Supplementary Table S1. Then, we explored the prognostic and diagnostic values of the CBX family in GBM, including differential gene expression analysis, survival analysis, and IDH mutation correlation status (26, 27). The procedures were conducted on R software (4.0.5), and the related R packages are exhibited in Supplementary Table S2.

### Human protein atlas

HPA is a tool with different protein expressions by immunocytochemistry in 20^*^12 cancers (https://www.proteinatlas.org) (28). We analyzed the differential expression of CBXs between GBM and normal brain tissues by website online tool.

### cBioPortal

cBioPortal was widely used for gene expression and alteration analysis based on the TCGA database (29). We first analyzed the different alterations of the CBX family in GBM (TCGA, Firehose Legacy). Moreover, we also explored the correlation between gene alteration and the prognosis of GBM patients. The mRNA expression z-scores relative to diploid samples were set at 1.5.

### Gene expression profiling interactive analysis (GEPIA) and cytoscape

We screened out the top 50^*^8 genes in GBM that were significantly associated with CBXs (Supplementary Figure S1) by GEPIA (http://gepia2.cancer-pku.cn/) and performed the GO and KEGG analyses by the Cytoscape software.

### Human tissue samples and cell lines

Primary glioblastoma (GBM) samples and normal brain tissues (NBTs) were obtained from the Department of Neurosurgery of the First Affiliated Hospital of Soochow University. The brain tumors were confirmed by neurosurgeons, radiologists, and pathologists based on a physical examination, neuroimaging, and histological examination. The human U87 and U251 glioma cell lines were purchased from the Shanghai Institutes for Biological Sciences and cultured in Dulbecco's modified Eagle medium (DMEM)/high glucose with 10% fetal bovine serum (FBS, Gibco, Carlsbad, CA, United States). All cells were maintained in a humidified incubator at 37 °C with 5% CO_2_.

### Immunohistochemistry staining and Immunofluorescent staining

Paraffin-embedded tissue sections were dewaxed (xylene, graded ethanol), peroxidase activity quenched (0.3% hydrogen peroxide), antigen-retrieved, and blocked (5% goat serum). Then, specific antibodies (CBX7: ab21873, 1:1,000 dilutions, CBX8: ab70796, 1:1,000 dilutions, Ki67: ab15,580, 1μg/ml, all purchased from Abcam, United Kingdom) covered the sections at 4°C overnight. Subsequently, specific secondary antibodies (1:100 dilutions, ZSGBBio, China) covered the sections for 1 h at 37°C, then were immersed in ABCperoxidase with diaminobenzidine (ZSGBBio, China), and counterstained with Mayer hematoxylin for 2 min (Solarbio, China). A microscope (Nikon, Tokyo, Japan) was used to observe the staining signals, and ImageJ Pro (Media Cybernetics, Rockville, Maryland, United States) was used by a technician (blinded to the experimental groupings) for statistical analysis.

The glioma cells that had undergone various interventions were fixed in 4% paraformaldehyde and permeabilized with 0.1% Triton-X for 5 min. Then, the glioma cells were incubated with primary antibodies (Ki67: ab15580, 1μg/ml, Abcam, United Kingdom) at 4°C overnight and related fluorescence-conjugated secondary antibody for 2 h at room temperature. Finally, the glioma cells were covered with a 2-(4-Amidinophenyl)-6-indolecarbamidine dihydrochloride (DAPI) solution and observed and analyzed using fluorescence microscopy (Nikon, Tokyo, Japan). A quantitative analysis was conducted by an observer who was blinded to the experimental groups.

### Transfection of lentivirus *in vitro*

A lentiviral packaging kit was purchased from GeneChem (Shanghai, China) to generate stable CBX8-knockdown (CBX8 siRNA) and CBX7-overexpression (CBX7 cDNA) glioma cell lines. According to the manufacturer's protocol, the hU6-MCS-CMV-EGFP lentiviral vectors encoding CBX8 small hairpin RNA (shRNA) or nontargeting shRNA were transfected with U87 and U251 glioma cells using HitransG A (REVG003, GeneChem, China). The cDNA of CBX7 was inserted into the lentiviral Ubi-MCS-3FL AG-CMV-EGFP vector that was sequentially transduced into the U87 and U251 cells (noncoding cDNA was set as control). CBX8-knockdown and CBX7-overexpression cell models were generated as described above. All the transfection efficiency was confirmed by fluorescence scanning and Western blot.

### Western blot

Cell protein samples were extracted, assessed (BCA kit, Beyotime, China), balanced, and denatured (with loading buffer, 100°C). Then the samples were added to a glue plate for electrophoresis and transferred to the membrane. After being sealed with milk, it was incubated with primary antibodies (MMP2, 1:1,000, Affinity, China; MMP9, 1:1,000, Affinity, China; CBX7, 1:1,000, Abcam, United Kingdom; CBX8, 1:1,000, Abcam, United Kingdom) at 4°C overnight and secondary antibody the next day. The band was exposed *via* the Enhanced Chemiluminescence (ECL) Kit (Beyotime) and quantified *via* the Image J Software (NIH, Bethesda, MD, United States) by a technician (blinded to the experimental groupings) for statistical analysis.

### Wound-healing assay

The U87 and U251 glioma cells were used to conduct the wound-healing assay with different interventions. The cell lay reached the appropriate confluence, and we scratched it with a 10-μl pipette tip. Then the cells were gently washed with PBS and cultured in a serum-free medium. The wound gap was photographed at 0 h and 24 h after the scratch, and measured by the Image J Software (NIH, Bethesda, MD, United States) to detect their difference in migration ability by a technician (blinded to the experimental groupings) for statistical analysis.

### Orthotopic glioma model *in vivo*

U87 cells, 3 x 10^5^, transfected with CBX7 cDNA or CBX8 shRNA, were subcutaneously injected into female nude mice (4–6 weeks). The tumor was photographed and measured at 0 h and 28 days after injection using the formula by a technician (blinded to the experimental groupings): volume (mm^3^) = length^*^width^*^height.

### Statistical methods

The data were statistically analyzed using GraphPad Prism 8 (GraphPad, San Diego, CA, United States) and presented as the mean ± standard deviation (SD). Data from two groups were analyzed by Student's unpaired two-sided *t*-test. Other statistical comparisons between more than two groups were performed using one-way ANOVA and *post hoc* least significant difference tests for multiple comparisons. A *p* < 0.05 was considered statistically significant for all statistical analyses.

## Results

### CBX2/3/5/8 were elevated and CBX6/7 were reduced in pan-cancers

**Figure 1:**
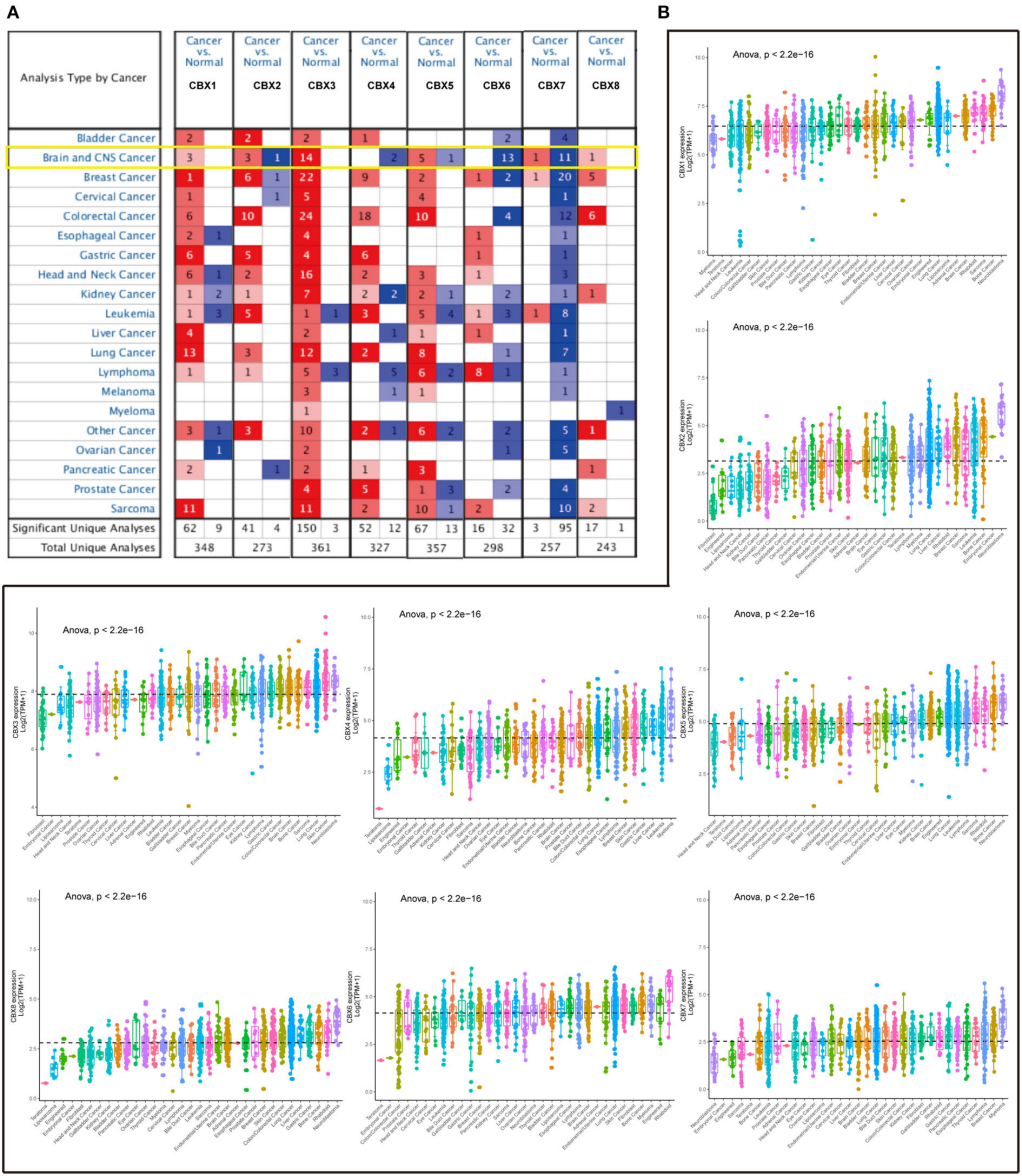
mRNA expression of CBX family in different cancers from tissues to cell lines. **(A)** Transcriptional expression of CBXs in 20 different types of cancers tissues (ONCOMINE). The difference in transcriptional expression was analyzed by Student's *t*-test. The cut-off of *p*-value was below 0.05. Fold changes should be beyond 1.5, and gene ranks should be in the top 10%. The yellow box emphasized the Brain and CNS cancers. **(B)** The difference of each CBX member at mRNA level in cell lines (CCLE) of 33 various cancers. Statistical comparisons were performed using one-way ANOVA and *post hoc* least significant difference tests for multiple comparisons. The dashed line represented the mean of the expression in the above 33 cancers.

To explore the roles of the CBX family in various cancers, we visualized the mRNA expression by ONCOMINE. As shown in Figure 1A, CBX1-3, CBX5, and CBX8 were significantly elevated in the brain and CNS cancers. However, CBX4, CBX6, and CBX7 were decreased compared to the normal samples. Further, we also explored the expression of the CBX family in the cell lines of pan-cancers *via* the cBioPortal. The CBX family exhibited high expression at the mRNA level similar to other cancers (Figure 1B). As shown in Table 1, the overexpression of CBX1 in GBM was up to 2.021-fold-change (30). Compared to normal tissues, the fold-change of CBX3 in GBM was > 1.6, as confirmed by seven GBM databases (30–34), including TCGA. Furthermore, CBX8 in GBM tissues presented a 1.928-fold-change more than that in normal tissue (33). Together, these data suggested the essential roles of CBX family in pan-cancers, especially in brain cancers.

**Table 1:** Changes of CBX family at mRNA and transcription level in GBM by ONCOMINE.

	**GBM vs. brain**	**Fold change**	***P*-value**	***T*-test**	**References**
CBX1	Glioblastoma	2.021	0.005	3.287	Shai Brain (30)
CBX3	Glioblastoma	3.130	6.59E-12	11.807	Shai Brain (30)
	Glioblastoma	1.907	2.71 E-5	7.124	TCGA Brain
	Glioblastoma	4.024	6.36 E-13	29.158	TCGA Brain
	Glioblastoma	2.002	3.19 E-16	21.975	Murat Brain (31)
	Glioblastoma	1.982	4.95 E-13	8.820	Sun Brain (32)
	Glioblastoma	2.369	0.012	4.653	Liang Brain (33)
	Glioblastoma	1.647	0.001	4.273	Bredel Brain 2 (34)
CBX8	Glioblastoma	1.928	1.38 E-10	7.163	Sun Brain (32)

*GBM, Glioblastoma; CBX, Chromobox*.

### The differential expressions of CBXs at mRNA level in TGCA and CGGA GBM database

**Figure 2:**
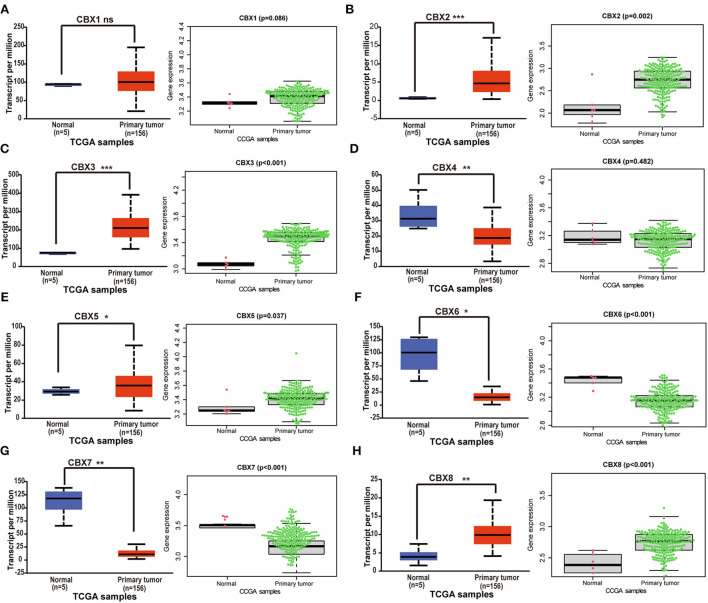
The differential expressions of CBX family at mRNA level in TCGA and CGGA. **(A-H)** The mRNA expression difference of CBXs in GBM was analyzed in The Cancer Genome Atlas (TCGA) and the Chinese Glioma Genome Atlas (CGGA) databases. UALCAN provided 156 GBM samples for analysis (ns, no significance; *p < 0.05, **p < 0.01, ***p < 0.001). 218 GBM samples from CGGA were downloaded and analyzed *via* “survminer” R package. The *p*-value was set at 0.05.

GBM is the most malignant tumor in brain cancers (3). To verify the differential expression of CBXs at the mRNA level in GBM, TCGA and CGGA databases were both used. On the UALCAN, we analyzed the CBXs mRNA expression in the 156 primary GBM tissues. Compared to the normal samples, the UALCAN revealed that CBX2, CBX3, CBX5, and CBX8 were upregulated (Figures 2B,C,E,H), and CBX4, CBX6, and CBX7 were downregulated (Figures 2D,F,G). Moreover, we also downloaded the mRNA sequencings from the CGGA and further analyzed the mRNA expression in 218 GBM patients *via* the R Software with the “beeswarm” package. A similar result was also indicated in the CGGA database. CBX2, CBX3, CBX5, and CBX8 displayed a higher expression in GBM than the normal tissues (Figures 2B,C,E,H), while CBX6 and CBX7 (Figures 2F,G) presented a lower expression. In these two databases, CBX1 had no significant difference between GBM and normal tissues (Figure 2A). Together, these findings indicated that the CBX family presented differential expressions in GBM at the mRNA level, which might play a critical role in the tumorigenesis and progression of GBM.

### The differential expressions of CBXs at protein level in HPA database

**Figure 3:**
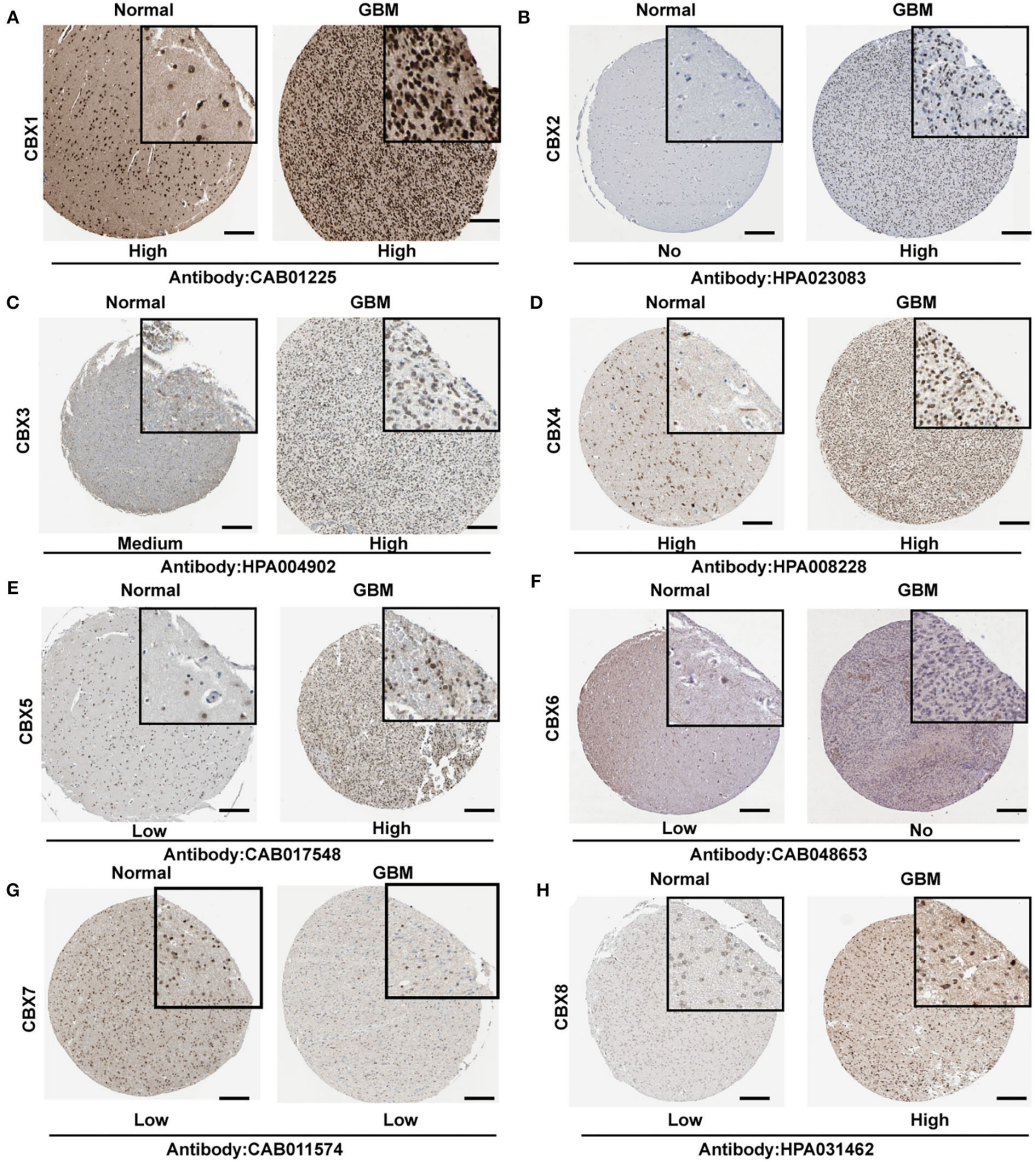
Representative immunohistochemistry images of CBX family in GBM and normal brain tissues from Human Protein Atlas (HPA). **(A–H)** The protein expression of each tissue was marked under the image. The scale bar represented 4 μm, and the image in the black square represented a 4x zoom. The antibody used in the staining was labeled below.

To further confirm the alteration of CBXs in the GBM tissue, we explored the protein levels of CBXs in HPA. CBX2, CBX3, CBX5, and CBX8 showed a higher expression in the GBM tissue than in normal tissue (Figures 3B,C,E,H). However, CBX1 and CBX4 in the GBM tissue (Figures 3A,D,G) showed a similar expression to normal tissue, whereas CBX6 presented a lower expression in GBM (Figure 3F). Moreover, CBX7 demonstrated a low expression both in GBM and normal tissue, which differed from the mRNA results in the TCGA and CGGA databases. Together, our findings indicated that the CBX family presented differential expressions in GBM at the protein level, primarily consistent with the above results at the mRNA level of GBM in the TCGA and CGGA databases.

### CBX2/6/7 were positively correlated with IDH mutation, and CBX1/2/4/5/8 was related to GBM subtypes

**Figure 4:**
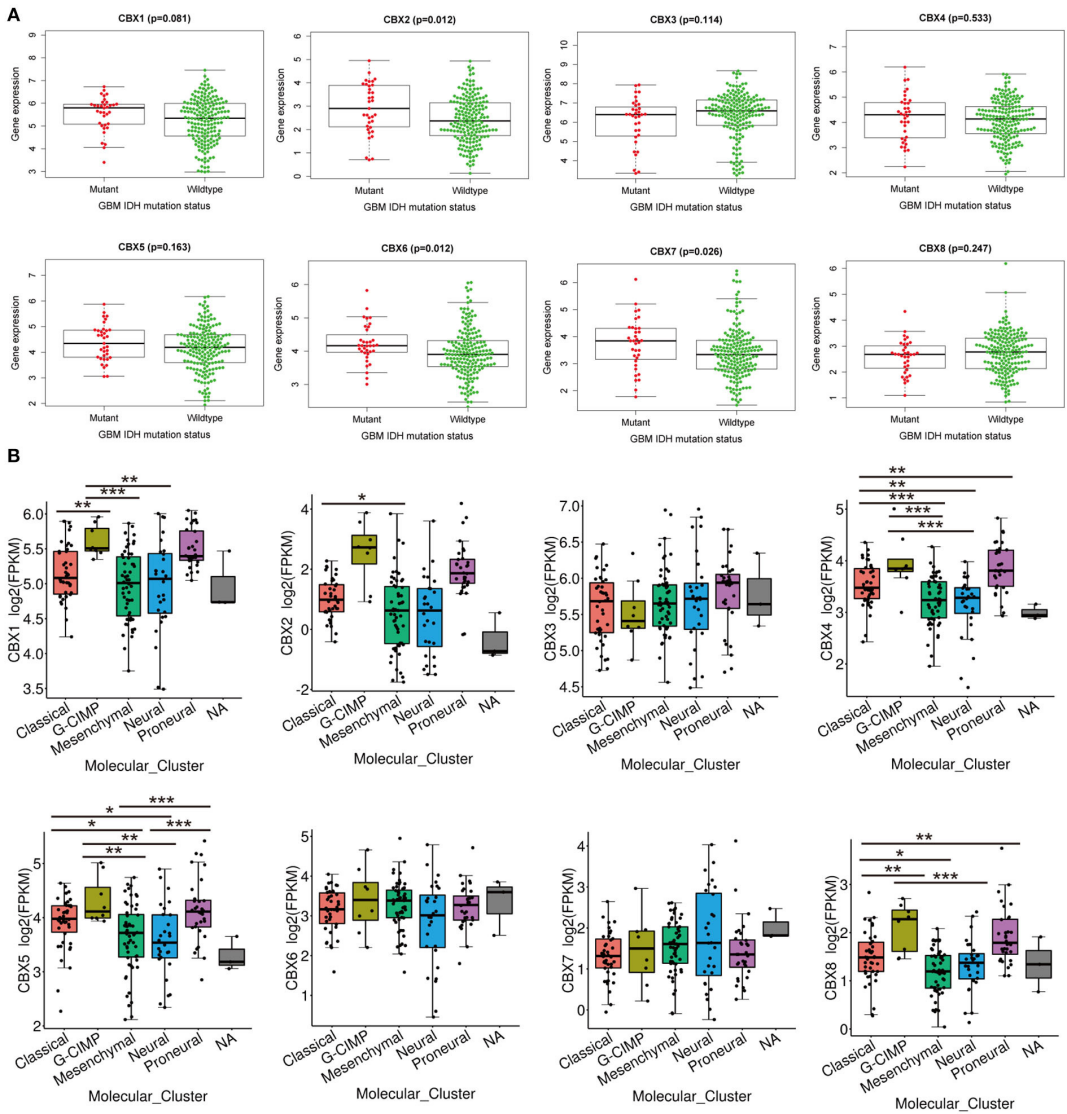
IDH mutation and molecular GBM subtypes were correlated with the mRNA expression of CBX family. **(A)** The difference in the mRNA expression of CBX family between IDH mutation (Red) and wildtype (Green) groups was explored in CGGA database by “survminer” R package. The *p*-value was set at 0.05. **(B)** Molecular subtypes in GBM, including Classical, Proneural, Neural, Classical, and Mesenchymal subtypes, were explored in differential mRNA expressions of CBX family by TCGA portal. (*p < 0.05, **p < 0.01, ***p < 0.001).

IDH mutation, a significant event in GBM progression, was widely used as a diagnostic and prognostic marker for GBM patients (35). We next explored the possible relationship between CBXs and IDH mutation in GBM from the CGGA data. We downloaded the 218-mRNA sequencing and conducted correlation analysis *via* the “beeswarm” R package. The result indicated that the higher expression of CBX2 (*p* = 0.012), CBX6 (*p* = 0.012), and CBX7 (*p* = 0.026) was closely related to IDH mutation, whereas the expressions of CBX1, CBX3, CBX4, CBX5, and CBX8 showed no relationship with the IDH mutation (Figure 4A). These data demonstrated that partial CBX family members were correlated with IDH mutation, suggesting a potential marker for predicting GBM survival and GBM IDH classification.

Additionally, GBM was divided into five different subtypes, including Proneural, Neural, Classical, and Mesenchymal types, based on the pathological features. These subtypes indicated different survival of GBM patients (36). Thus, we tested whether CBXs were associated with different GBM subtypes in a TCGA portal. As shown in Figure 4B, CBX1 showed significantly differential expression in GBM between G-CIMP and Classical, Mesenchymal, and Neural subtypes. CBX2 displayed a distinct difference in the Classical and Mesenchymal subtypes of GBM. CBX4 showed a substantial difference between the Classical subtype and Neural, Mesenchymal, and Proneural subtype. There was also a differential expression of CBX4 between the G-CIMP subtype and Mesenchymal, Neural subtype. Except for the groups between Proneural and Classical, G-CIMP, as well as Neural and Mesenchymal subtypes, all the rest of the groups displayed differential expressions of CBX5. CBX8 showed a notable difference in Classical and G-CIMP, Mesenchymal, Proneural subtypes, as well as G-CIMP and Neural subtypes. Together, these data demonstrated that the differential expression of CBXs was intimately related to GBM pathological subtypes, which may serve as valuable markers for GBM classification.

### CBX7 and CBX8 were independently associated with the survival time of GBM patients

**Figure 5:**
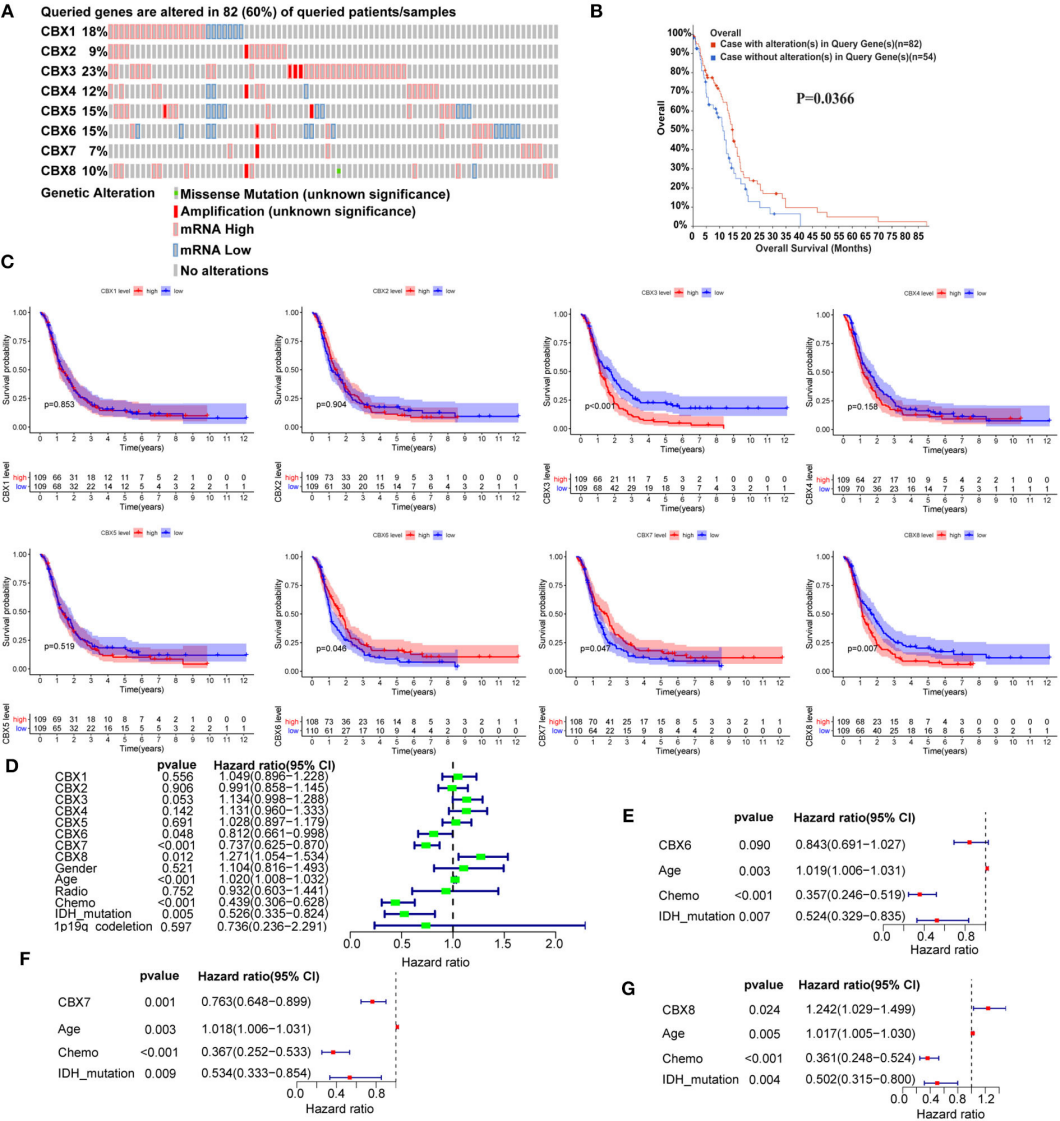
The prognostic value of CBX family in GBM patients. **(A)** The genetic mutation of CBX members in GBM patients was analyzed on cBioPortal (TCGA, Firehose Legacy). The z-scores of mRNA expression relative to diploid samples were set at 1.5. Except for the gray region (No alteration), the other colors represented different genetic mutations in these patients. **(B)** The prognostic value of alterations in CBX genes was analyzed by Kaplan-Meier analysis on cBioPortal. **(C)** The correlation between mRNA expression and GBM patients' survival was explored by Kaplan-Meier analysis in CGGA data. The red dash represented high mRNA expression, and the blue meant low expression. **(D)** The univariate Cox survival analysis was conducted to screen out the significant single factors that contributed to the survival of GBM patients. The *p*-value threshold was 0.5. **(E–G)** Further multivariate Cox survival analysis was used to confirm the independent prognostic values of the above significant single factors. The above Kaplan-Meier, univariate, and multivariate survival analyses using R software with the “survival” and “survminer” packages.

To determine the prognostic values of the CBX family, we explored the genetic alteration of CBXs in GBM (TCGA, Firehose Legacy) *via* the cBioPortal. A total high mutation rate (82/136, 60%) of CBXs was detected in the GBM patients. In detail, the CBX mutation was widespread and ranged from 7% to 23% (CBX 1: 18%; CBX 2: 9%; CBX 3: 23%; CBX 4: 12%; CBX 5: 15%; CBX 6: 15%; CBX 7: 7%; CBX 8: 10%) (Figure 5A). Moreover, the GBM patients with CBXs alteration presented better survival than those without CBX alteration. Figure 5B, suggesting an important role of CBXs alteration in the prognosis of GBM patients.

Furthermore, we conducted the comprehensive survival analysis of CBXs in GBM of CGGA data using R software with the “survival” and “survminer” packages. We found that GBM patients with high expression of CBX3 and CBX8 displayed a poorer survival, while those with increased CBX6 and CBX7 indicated a better survival (Figure 5C). To further confirm the independent diagnostic role of CBXs in GBM, we performed Cox survival regression analysis, including the CBX family, gender, age, radiotherapy, chemotherapy, IDH mutation, and 1p19q codeletion. The univariate analysis results indicated that CBX6, CBX7, CBX8, age, chemotherapy, and IDH mutation played a prognostic role in GBM (Figure 5D). Then the CBX members, age, chemotherapy, and IDH mutation were further included to perform the multivariate analysis. The results indicated that CBX7 and CBX8 showed independent prognostic values in GBM (Figures 5E–G). Together, these data revealed that CBX7 and CBX8 were the independent factors contributing to the survival of GBM patients.

### Elevated CBX7 and decreased CBX8 suppressed the proliferation and invasion of tumor cells in GBM

**Figure 6:**
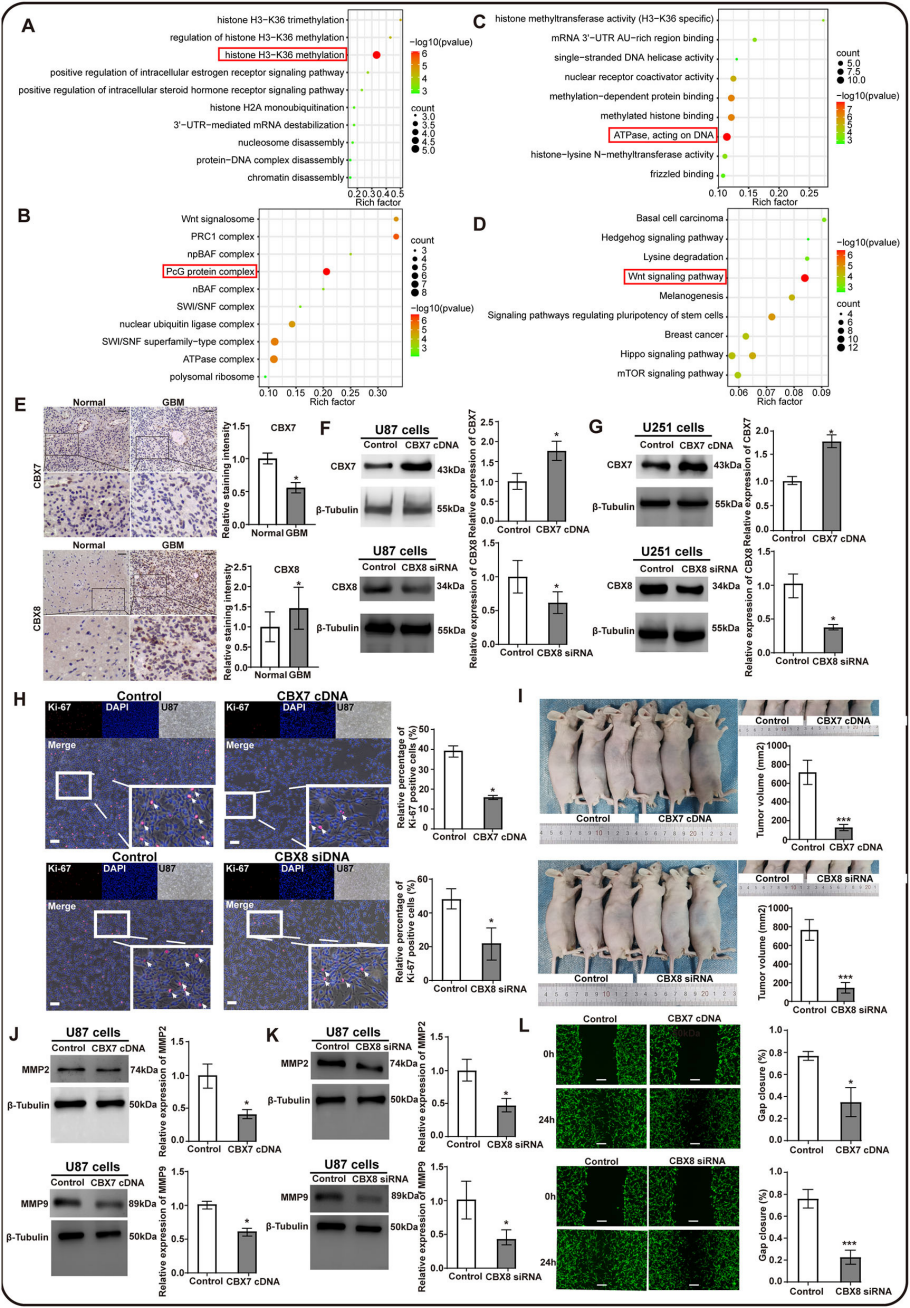
The potential function prediction and validation of CBX family in proliferation and invasion of glioma cells. The top 50*8 genes associated with CBX members in GBM were screened out by GEPIA and used to conduct the GO and KEGG analyses by Cytoscape. **(A)** Biological process (BP), **(B)** Cellular components (CC), **(C)** Molecular functions (MF), and **(D)** the KEGG pathways influenced by CBX family. The red box emphasized the functions that had the most count and significant difference in these analyses. **(E)** Clinical validation of CBX7 and CBX8 through immunochemistry staining in tumor-adjacent normal and GBM tissues. The black scale bar represented 4 μm. *N* = 3. (*p < 0.05). **(F,G)** Western blots were performed to confirm the effectiveness of lentivirus transfection in U87 and U251 glioma cells. β-tubulin was set as the loading control. *N* = 3. (*p < 0.05). **(H)** Ki-67 immunofluorescent staining was performed to evaluate the effect of CBX7 and CBX8 on the proliferation capacity in glioma cells. The white scale bar represented 20 μm. **(I)** The orthotopic U87 glioma model in nude mice measured the proliferation of glioma cells with intervention on CBX7 and CBX8. The subcutaneous tumor was photographed and measured at 0 h and 28 days after injection using the formula: volume (mm^3^) = length*width*height. *N* = 3. **(J,K)** Western blots were performed to assess the invasion-related markers by detecting the MM2 and MMP9. β-Tubulin was set as the loading control, *N* = 3, (*p < 0.05). **(L)** The wound-healing assay explored the invasion abilities of U87 glioma cells with different interventions on CBX7 and CBX8 compared to the control. The gap was measured at 0 h and 24 h after the scratch. The white scale bar represented 4 μm. (*p < 0.05, **p < 0.01, ***p < 0.001).

The CBX family was a critical component of epigenetic regulators that repressed the transcription of target genes through chromatin modification, contributing to tumorigenesis and tumor progression (37, 38). However, the exact function of CBXs in GBM remained unclear. Thus, we screened out the top 50^*^8 genes significantly associated with CBX members (Supplementary Figure S1) in GBM from the TCGA data and performed the GO and KEGG analyses by the Cytoscape software. The results of the biological process (BP) and cellular component (CC) indicated that the CBX family was related to histone H3–K36 methylation and exerted an effect on the PcG protein complex, as indicated by the red box (Figures 6A,B). Moreover, the molecular function analysis demonstrated that the function of the CBX family was associated with ATPase, which acted on DNA (Figure 6C). Notably, CBXs exerted biological effects on the tumor cells through the classical Wnt signaling pathway (Figure 6D), which was confirmed to be related to the proliferation and invasion of tumor cells during tumorigenesis and progression (39–41). These findings suggested that the CBX family possibly promoted malignant tumor phenotype *via* epigenetically regulating target genes.

To further confirm the role of CBXs in the GBM, we tested the hypothesis that CBXs might play significant roles in GBM propagation and invasion. Immunohistochemistry staining of CBX7 and CBX8 was performed on the clinical GBM tissues in our medical center due to their independent role in the GBM prognosis. We found similar results to the HPA database that CBX7 in the GBM tissues also showed a lower expression than their adjacent normal tissues, whereas CBX8 presented a higher expression in the GBM tissues (Figure 6E), suggesting that CBX7 functioned as an anti-oncogene and CBX8 as an oncogene in the tumorigenesis and progression of GBM. Next, based on the gene regulation technology using lentivirus transfection, we established two models of CBX7-upregulated expression and CBX8-downregulated expression in the U87 and U251 glioma cells, respectively. Western blots were performed to confirm the effectiveness of transfection (Figures 6F,G). The results indicated that CBX7 was overexpressed, and CBX8 was knockdown significantly after lentivirus transfection compared to the control.

Next, we explored the effect of CBX7 and CBX8 on the proliferation capacity in these two glioma cells. We found that the glioma cells presented a reduced proliferation capacity after gene intervention on CBX7 and CBX8, as indicated by the Ki67 immunostaining *in vitro* (Figure 6H; Supplementary Figure S2A). Moreover, after orthotopical injection of various glioma cells with lentivirus transfection for 28 days *in vivo*, the tumor volume presented a significantly lower volume in glioma with overexpressed CBX7 /downregulated CBX8 than in the control groups, as indicated in Figure 6I. These data demonstrate that CBX7 and CBX8 exert an effect on the propagation ability of the glioma cells. Additionally, we also evaluated the effect of CBX7 and CBX8 on the invasion ability of glioma cell cells by detecting invasion-related markers of matrix metalloproteinase 2 (MMP2) and MMP9, and further confirmed the corresponding phenotypes of glioma cells by scratch wound-healing assay. The result indicated that overexpressed CBX7 and downregulated CBX8 in glioma cells showed a decreased expression of MMP2 and MMP9 (Figures 6J,K; Supplementary Figures S2B,C) and displayed significantly lower invasion abilities in scratch wound-healing assay when compared to the control (Figure 6L; Supplementary Figure S2D). These data suggested that CBX7 and CBX8 could promote the cell proliferation and invasion of GBM, leading to poor survival of GBM patients.

## Discussion

CBX family, a critical component of epigenetic regulators, has been confirmed to be involved in tumor propagation, invasion, and recurrence (42, 43), which was closely related to the prognosis of patients. However, the roles of the CBX family in GBM are still inconclusive. In this study, we comprehensively analyzed the differential expression of CBXs at the mRNA and protein levels in GBM using the TCGA and CGGA databases. Moreover, we further explored the relationship between CBXs and clinical GBM classification, including IDH mutation and GBM subtypes, which contributes to the diagnosis and prognosis of GBM. We pinpointed that CBX7 and CBX8 exhibited independent prognostic values in GBM by analyzing the clinical data. CBXs were found to be closely related to the histone H3-K36, PcG protein complex, ATPase, and Wnt pathway in GBM. Finally, we confirmed the roles of CBX7 and CBX8 in the proliferation and invasion of glioma cells *in vivo* and *in vitro* experiments, which provided a promising strategy for GBM treatments.

In this study, we confirmed the expression of CBX7 in the clinical GBM tissues. Compared to the normal group, CBX7 presented a lower expression in the GBM. This conclusion was further confirmed by previous results (15). They found that CBX7 was downregulated in GBM tissues. Furthermore, we also verified the prognostic role of CBX7 in the CGGA database with univariate and multivariate analyses, which was similar to previous results (14, 44). The glioma cells with the overexpression of CBX7 could improve the survival of implanted mice *in vivo*. These findings were related to the critical role of CBX7 in the proliferation and invasion of glioma cells, as indicated in our experiments as well as those conducted by others *in vivo* and *in vitro* (14, 15, 44).

Contrary to the role of CBX7 in GBM, CBX8 was another independent prognostic factor in our study. We found that CBX8 displayed a higher expression in GBM than the normal tissue at the mRNA and protein levels in the TCGA and CGGA data. Moreover, the patients with an overexpression of CBX8 presented poorer survival, as evidenced by the corresponding clinical data. These data were mostly in line with previous results using different glioma databases (16, 17). They found that the higher expression of CBX8 in glioma indicated a more dismal prognosis of GBM due to its positive role in the propagation and invasion of glioma cells. In our studies, we confirmed the same effect of CBX8 on the glioma cells *in vivo* and *in vitro*. Importantly, we also validated its independent prognostic role in the GBM patients *via* univariate and multivariate analyses. These findings demonstrated more convincible results on the oncogenic role of CBX8 in the GBM.

Additionally, we also explored the differential expressions of other CBXs between GBM and normal brain tissue in the TCGA and CGGA data. The results indicated that CBX2, CBX3, CBX5, and CBX8 exhibited higher expression than normal tissue, while CBX6 and CBX7 presented a lower face in the Kaplan-Meier analysis. These results were partially consistent with a previous study in a different online database (13, 45). They concluded differently that CBX3, CBX6, and CBX8 in GBM displayed a prognostic value based on a single Kaplan-Meier analysis. However, we further confirmed that CBX7/8 rather than CBX3/6 presented independent prognostic roles in GBM by performing univariate and multivariate analyses.

IDH mutation and GBM subtypes, including Classical, CIMP, Mesenchymal, Proneural, and Neural (36), were confirmed to be significant markers in the diagnosis and prognosis of GBM. Thus, these two markers and CBXs were included in our study to perform the correlation analysis. We found that most CBXs were significantly correlated with IDH mutation and GBM subtypes. Particularly, CBX7 was related to the state of IDH mutation while CBX8 was in relation to GBM subtypes like the groups between Classical and G-CIMP/Mesenchymal/Proneural subtypes, as well as G-CIMP and Neural subtypes. These data suggested that CBXs may serve a potential role in predicting the subtypes and prognosis of GBM.

Our study comprehensively analyzed the roles of CBXs in GBM at the mRNA and protein levels in a double-cohorts validation. We finally screened out CBX7 and CBX8, which functioned as the independent prognostic factors in GBM by the Kaplan-Meier and univariate/multivariate analyses. Furthermore, we explored and confirmed the roles of CBX7 and CBX8 in the proliferation and invasion of glioma cells *in vivo* and *in vitro*. These data provided potential but promising targets for the GBM treatment. However, despite figuring out the possible Wnt pathway by which the CBX family mediated the phenotype of GBM, we did not further confirm the relationships between the CBXs and Wnt pathway. In the future, more evidence would be needed to clarify their complex interaction. Anyway, this study, at least partially, revealed a preliminary mechanism of CBX7/8 in the GBM phenotype and provided a promising strategy for GBM treatment.

## Data availability statement

The datasets presented in this study can be found in online repositories. The names of the repository/repositories and accession number(s) can be found in the article/Supplementary material.

## Ethics statement

The studies involving human participants were reviewed and approved by the Ethics Committee of the First Affiliated Hospital of Soochow University. The patients/participants provided their written informed consent to participate in this study.

## Author contributions

Conceptualization and data curation: Z-QZ and G-QY. Methodology, editing and revising, and writing-original draft preparation: Z-QZ, G-QY, and N-LK. Formal analysis and experimental investigation: Z-QZ, G-QY, Q-QN, and G-GZ. Supervision: Z-QZ and ZW. All authors have read and agreed to the published version of the manuscript.

## Conflict of interest

The authors declare that the research was conducted in the absence of any commercial or financial relationships that could be construed as a potential conflict of interest.

## Publisher's note

All claims expressed in this article are solely those of the authors and do not necessarily represent those of their affiliated organizations, or those of the publisher, the editors and the reviewers. Any product that may be evaluated in this article, or claim that may be made by its manufacturer, is not guaranteed or endorsed by the publisher.
