# Supplementary material for: Chromobox 7/8 serve as independent indicators for glioblastoma via promoting proliferation and invasion of glioma cells
Source: Front Neurol. 2022 Aug 11;13:912039. doi: 10.3389/fneur.2022.912039 (PMC9403790; doi:10.3389/fneur.2022.912039)
Supplement: Supplementary file 2 [file Data_Sheet_1.docx]

**Supplemental Information**

Chromobox 7/8 served as independent indicators for glioblastoma via promoting proliferation and invasion of glioma cells

Zong-Qing Zheng^1†^, Gui-Qiang Yuan^1,2†^, Na-Ling Kang^3^, Qian-Qian Nie^1^, Guo-Guo Zhang^1^, Zong Wang^1^

^1^ Department of Neurosurgery & Brain and Nerve Research Laboratory, The First Affiliated Hospital of Soochow University, Suzhou, Jiangsu Province, China.

^2^ Department of Neurosurgery, Changshu Second People's Hospital, Suzhou, China.

^3^ Liver Center, The First Affiliated Hospital, Fujian Medical University, China, 350005.

^†^These authors have contributed equally to this work and share the first authorship.

**Content:**

1. Supplemental Figures (S1, S2)
2. Supplemental Figures legends (S1, S2)
3. Supplemental Tables (S1, S2)
4. **Supplemental Figures:**

Figure S1.


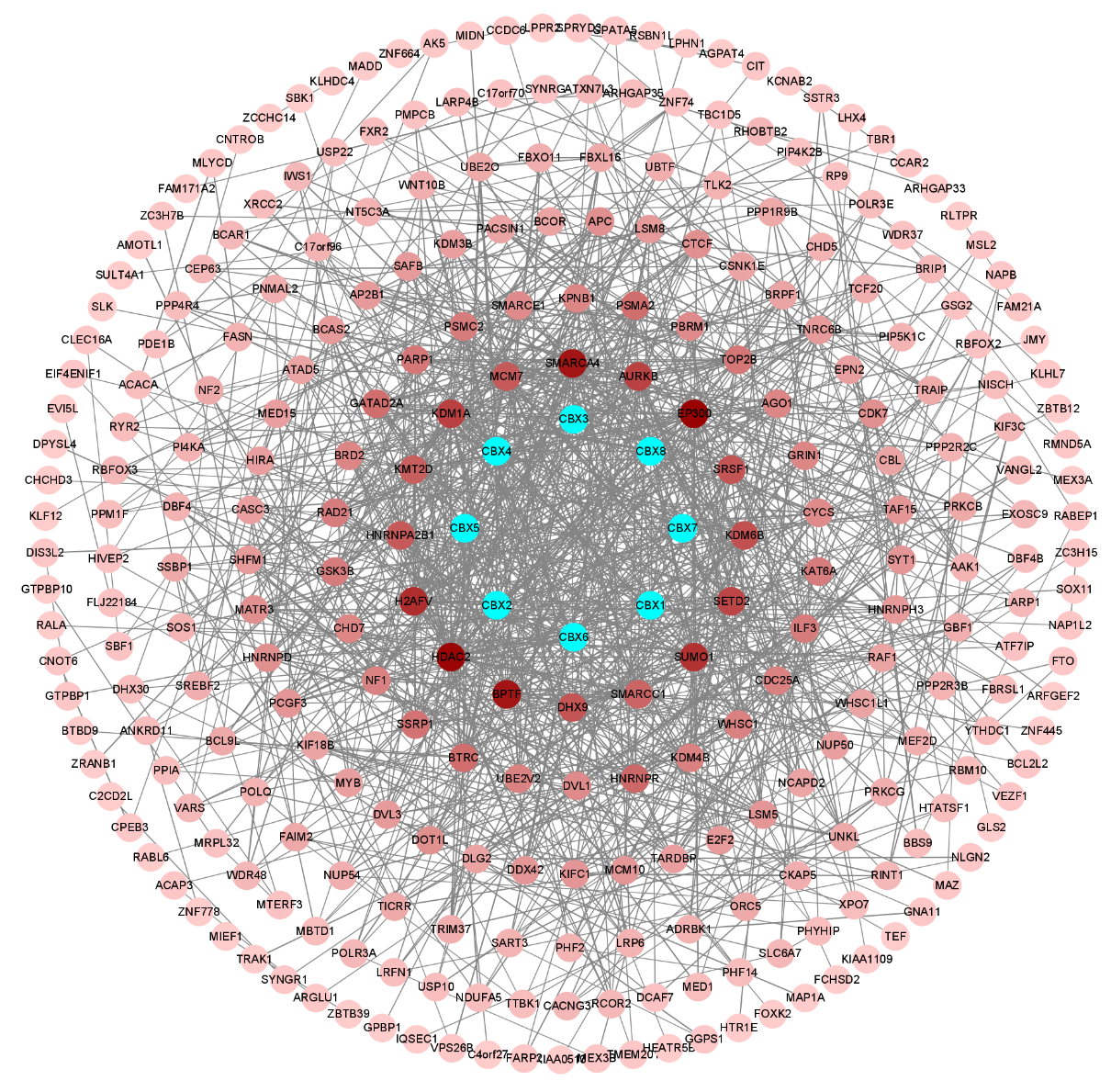


Figure S2.


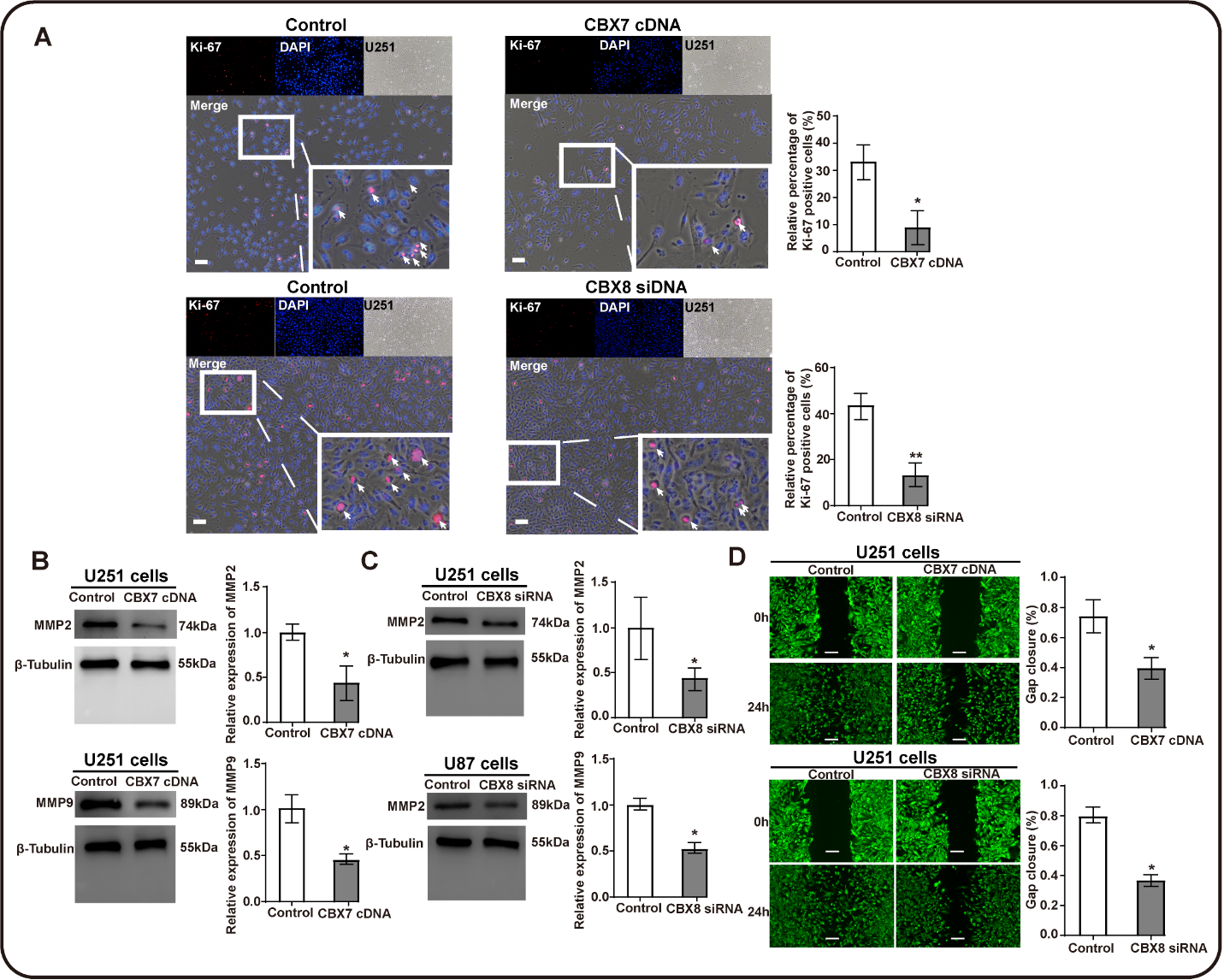


1. **Supplemental Figures legends:**

**Figure S1. The top 50*8 genes were significantly associated with eight CBX members in GBM by GEPIA**. The blue dots represented the CBX family members and the red dots meant the correlated genes. The gray lines indicated the correlation and the density of the red showed the strength of the association.

**Figure S2. The validation of CBX family in proliferation and invasion of U251 glioma cells**. **(A)** Ki-67 immunofluorescent staining was performed to evaluate the effect of CBX7 and CBX8 on the proliferation capacity in U251 glioma cells. The white scale bar represented 20 μm. **(B-C)** Western blots were performed to assess the invasion-related markers by detecting the MM2 and MMP9. β-Tubulin was set as the loading control, N = 3, (*p < 0.05). **(D)** The wound-healing assay explored the invasion abilities of U251 glioma cells with different interventions on CBX7 and CBX8 compared to the control. The gap was measured at 0 h and 24 h after the scratch. The white scale bar represented 4 μm. (*p < 0.05, **p < 0.01)

1. **Supplemental Tables:**

**Table S1. Basic characteristics of 218 GBM patients in CGGA database.**

| **Variables** | |
| --- | --- |
| Gender( Male/female) | 133/85 |
| Age(years, Mean±SD) | 51.06±12.97 |
| Survival time（days, Median） | 717(19-4435) |
| Censor | N=218 |
| Radio-therapy | N=211 |
| Chemo-therapy | N=210 |
| IDH mutation | N=211 |
| IDH_mutation_status | 192 |
| Expression of Chromobox 1, 2, 3, 4, 5, 6, 7, 8 | N=218 |

GBM: Glioblastoma, SD: standard deviation.

**Table S2. R** **programming language used R packages in this study.**

R programming language version 4.0.5.

| **Analysis** | **R packages** |
| --- | --- |
| mRNA expression analysis in CGGA | "beeswarm" |
| IDH mutation analysis |  |
| Kaplan-Meier analysis | "survival", "survminer" |
| Cox analysis and multi-Cox analysis |  |
